# Flowers

**Published:** 1875-06

**Authors:** 


Flowers were created to gladden the heart of man, cheer his lonely hours, afford him instruction, and give him perpetual themes for joyful admiration. The cultivation of flowers, besides being a healthful exercise for young ladies, softens the disposition and refines the taste. You will almost invariably find that the woman who likes to cultivate these beauties of nature, is a kind and affectionate companion, and keeps a well-ordered household. It also gives a taste for the beautiful, and the mind will naturally pass to a love of all that is grand and sublime in nature. Even the Saviour drew some of His most excellent illustrations from the lilies of the field.

---

In the intercourse of social life, it is by little acts of watchful kindness recurring daily and hourly—and opportunities of doing kindness, if sought for are forever starting up—it is by words, by tones, by gestures, by looks, that affection is won and preserved. He who neglects these trifles, yet boasts that whenever a great sacrifice is called for, he shall be ready to make it, will rarely be loved. The likelihood is he will not make it; and if he does, it will be much rather for his own sake than for his neighbors.

---

On all sides, the opinion prevails that married life should begin at the high tide of indulgence in all that constitutes an elegant, fashionable, and wealthy home, or ought not to begin at all. The old idea of "love in a cottage" is put aside as a barbaric relic of the rude past. Nothing short of fashionable life amid elegant and luxurious surroundings, either with or without love, will meet the requirements of the refined present. This is all wrong.

